# Engineering HIV-1-Resistant T-Cells from Short-Hairpin RNA-Expressing Hematopoietic Stem/Progenitor Cells in Humanized BLT Mice

**DOI:** 10.1371/journal.pone.0053492

**Published:** 2012-12-31

**Authors:** Gene-Errol E. Ringpis, Saki Shimizu, Hubert Arokium, Joanna Camba-Colón, Maria V. Carroll, Ruth Cortado, Yiming Xie, Patrick Y. Kim, Anna Sahakyan, Emily L. Lowe, Munetoshi Narukawa, Fadi N. Kandarian, Bryan P. Burke, Geoff P. Symonds, Dong Sung An, Irvin S. Y. Chen, Masakazu Kamata

**Affiliations:** 1 Department of Microbiology, Immunology, and Molecular Genetics, David Geffen School of Medicine at University of California Los Angeles, Los Angeles, California, United States of America; 2 University of California Los Angeles AIDS Institute, Los Angeles, California, United States of America; 3 Division of Hematology-Oncology, David Geffen School of Medicine at University of California Los Angeles, Los Angeles, California, United States of America; 4 Calimmune, Inc., Los Angeles, California, United States of America; 5 University of California Los Angeles School of Nursing, Los Angeles, California, United States of America; 6 Department of Biology, California State University Northridge, Northridge, California, United States of America; 7 University of California Los Angeles Broad Stem Cell Research Center, Los Angeles, California, United States of America; University Hospital Zurich, Switzerland

## Abstract

Down-regulation of the HIV-1 coreceptor CCR5 holds significant potential for long-term protection against HIV-1 in patients. Using the humanized bone marrow/liver/thymus (hu-BLT) mouse model which allows investigation of human hematopoietic stem/progenitor cell (HSPC) transplant and immune system reconstitution as well as HIV-1 infection, we previously demonstrated stable inhibition of CCR5 expression in systemic lymphoid tissues via transplantation of HSPCs genetically modified by lentiviral vector transduction to express short hairpin RNA (shRNA). However, CCR5 down-regulation will not be effective against existing CXCR4-tropic HIV-1 and emergence of resistant viral strains. As such, combination approaches targeting additional steps in the virus lifecycle are required. We screened a panel of previously published shRNAs targeting highly conserved regions and identified a potent shRNA targeting the R-region of the HIV-1 long terminal repeat (LTR). Here, we report that human CD4^+^ T-cells derived from transplanted HSPC engineered to co-express shRNAs targeting CCR5 and HIV-1 LTR are resistant to CCR5- and CXCR4- tropic HIV-1-mediated depletion *in vivo*. Transduction with the combination vector suppressed CXCR4- and CCR5- tropic viral replication in cell lines and peripheral blood mononuclear cells *in vitro*. No obvious cytotoxicity or interferon response was observed. Transplantation of combination vector-transduced HSPC into hu-BLT mice resulted in efficient engraftment and subsequent stable gene marking and CCR5 down-regulation in human CD4^+^ T-cells within peripheral blood and systemic lymphoid tissues, including gut-associated lymphoid tissue, a major site of robust viral replication, for over twelve weeks. CXCR4- and CCR5- tropic HIV-1 infection was effectively inhibited in hu-BLT mouse spleen-derived human CD4^+^ T-cells *ex vivo*. Furthermore, levels of gene-marked CD4^+^ T-cells in peripheral blood increased despite systemic infection with either CXCR4- or CCR5- tropic HIV-1 *in vivo*. These results demonstrate that transplantation of HSPCs engineered with our combination shRNA vector may be a potential therapy against HIV disease.

## Introduction

Acquired immune deficiency syndrome (AIDS) caused by human immunodeficiency virus type 1 (HIV-1) infection remains one of the most important global public health threats [1]. Advances in anti-HIV-1 therapy such as highly active antiretroviral therapy (HAART) have markedly improved patient survival. However, most of these treatments are likely never to be curative and are limited by toxicity, cost, and, especially, the emergence of resistant viral strains [2]. Thus, alternative methods that decrease or eliminate the need for such lifelong continuous treatments and combat HIV-1 resistance to current therapies are greatly desired. Gene therapy which could provide lifelong therapeutic interventions with one or few administrations is a potentially promising approach [3]–[7].

HIV-1 utilizes the CD4 receptor and, primarily, chemokine receptors CXCR4 and CCR5, as co-receptors that are crucial for viral entry into host cells [8]–[10]. As such, inhibition of the co-receptor-virion interaction has elicited much therapeutic interest [8], [11], [12]. Landmark population genetic and molecular studies demonstrated that individuals homozygous for a defective CCR5 gene, CCR5Δ32, are protected from HIV-1 infection [13]–[15]. Recently, an HIV-1 positive individual with concurrent acute myeloid leukemia (AML) was treated by transplant of allogeneic hematopoietic stem progenitor cells (HSPCs) isolated from a CCR5Δ32 homozygous donor. Remarkably, the CCR5Δ32 donor cells nearly completely replaced the recipient's cells within a rapid 61 days and HIV-1 virus has remained undetectable in the patient despite discontinuation of HAART for more than 4 years [16], [17]. This striking evidence supports the idea that stable down-regulation of CCR5 expression could result in reduced viral load and prevent the progression to AIDS in HIV-1 infected patients. However, the difficulty of finding a human leukocyte antigen matched CCR5Δ32 homozygous donor considerably limits widespread use of this strategy.

RNA interference (RNAi) is a highly evolutionarily conserved mechanism of post-transcriptional gene silencing that can be triggered by small interfering RNAs (siRNAs) [18]. In addition to its experimental utility in loss-of-function studies, RNAi emerges as a potentially powerful therapeutic approach towards human diseases. We and others have developed lentiviral vectors encoding short hairpin RNA (shRNA), commonly driven by RNA polymerase III promoters H1 or U6, which are processed by cellular machinery into siRNA for stable inhibition of HIV-1 co-receptors and HIV-1 gene expression [19]–[26]. We previously demonstrated efficient knock-down of CCR5 expression via H1 promoter-driven expression of a highly potent CCR5-specific shRNA (sh1005) in human primary T-cells [27] and macrophages [28], resulting in strong inhibition of HIV-1 infection *in vitro*. Importantly, we found it necessary to screen extensively for shRNAs that maintain potency at low expression levels to avoid cytotoxic effects associated with shRNA overexpression [27].

Use of various humanized small animal models, where immunocompromised mice are reconstituted with either HSPCs or differentiated T-cells to confer susceptibility to HIV-1 infection, have facilitated preclinical assessments of *in vivo* efficacy of various CCR5-targeted HIV-1 therapies. While several humanized mouse model studies have focused on systemically delivered methods such as CCR5-specific RNAi inducers, coupled with aptamers [29], nanoparticles [30], [31], or peptides [32], as well as small molecule CCR5 antagonists [33], [34] which require repeated doses, potentially longer-lasting strategies using genetically modified HSPCs have also been explored *in vivo*. While others have employed zinc-finger nuclease-mediated genome editing [35], we have demonstrated continuous RNAi-mediated down-regulation of CCR5 expression via vector-transduced HSPC transplantation [36]. To our knowledge, we reported the first application of the humanized bone marrow/liver/thymus (hu-BLT) mouse model [37], [38] as an *in vivo* AIDS gene therapy model.

The hu-BLT mouse model provides robust peripheral reconstitution of human T-cells, B-cells, and macrophages and importantly, unlike other models, efficient repopulation of many lymphoid tissue compartments including highly CCR5-expressing bone marrow and gut-associated lymphoid tissue (GALT), a primary target site of CCR5-tropic HIV-1 infection [39]. Hence, the hu-BLT mouse has become a model of choice to investigate HIV-1 infection and pathogenesis. Previously, we showed in hu-BLT mice successful engraftment of transplanted fetal-liver-derived CD34^+^ (FL-CD34^+^) cells transduced with the sh1005-encoding vector and differentiation into CCR5-down-regulated T-cells and monocytes/macrophages in peripheral blood and systemic lymphoid organs [36]. Similar observations were seen in our nonhuman primate rhesus macaque studies [21]. Cells transduced with this vector showed excellent protection against CCR5 (R5)-tropic [21], [36], but not CXCR4 (X4)-tropic [36], viral strains. Therefore, CCR5 down-regulation, although promising against infection by R5-tropic viral strains, would be ineffective against pre-existing X4-tropic and dual tropic strains or the emergence of viral escape mutant strains, necessitating the incorporation of additional therapeutic reagents.

To confer protection against HIV-1 strains unimpeded by sh1005-mediated CCR5 down-regulation, we evaluated the anti-HIV-1 effects of selected previously published shRNAs targeting conserved regions of the HIV-1 genomic sequence. After screening for candidates with high anti-viral effects at low shRNA expression levels, we selected sh516, which targets the long terminal repeat (LTR) R region of HIV-1. Following extensive vector characterization *in vitro*, we evaluated the *in vivo* reconstitution and stability of HSPCs engineered with our novel sh1005/sh516 combination vector and assessed conferred anti-viral potency of transplanted HSPC-derived T lymphocytes. Here, we report that transplantation of sh1005/sh516-transduced HSPCs resulted in efficient engraftment, stable marking in resultant hematopoietic lineages and potent inhibition of HIV-1-mediated depletion of modified CD4^+^ T-cells *in vivo*. This work describes a novel and safe combination vector which may provide both potent anti-viral inhibition and high reconstitution efficiencies for effective control of HIV infection.

## Results

### Selection of a Single Potent Anti-HIV-1 shRNA

**Figure 1:**
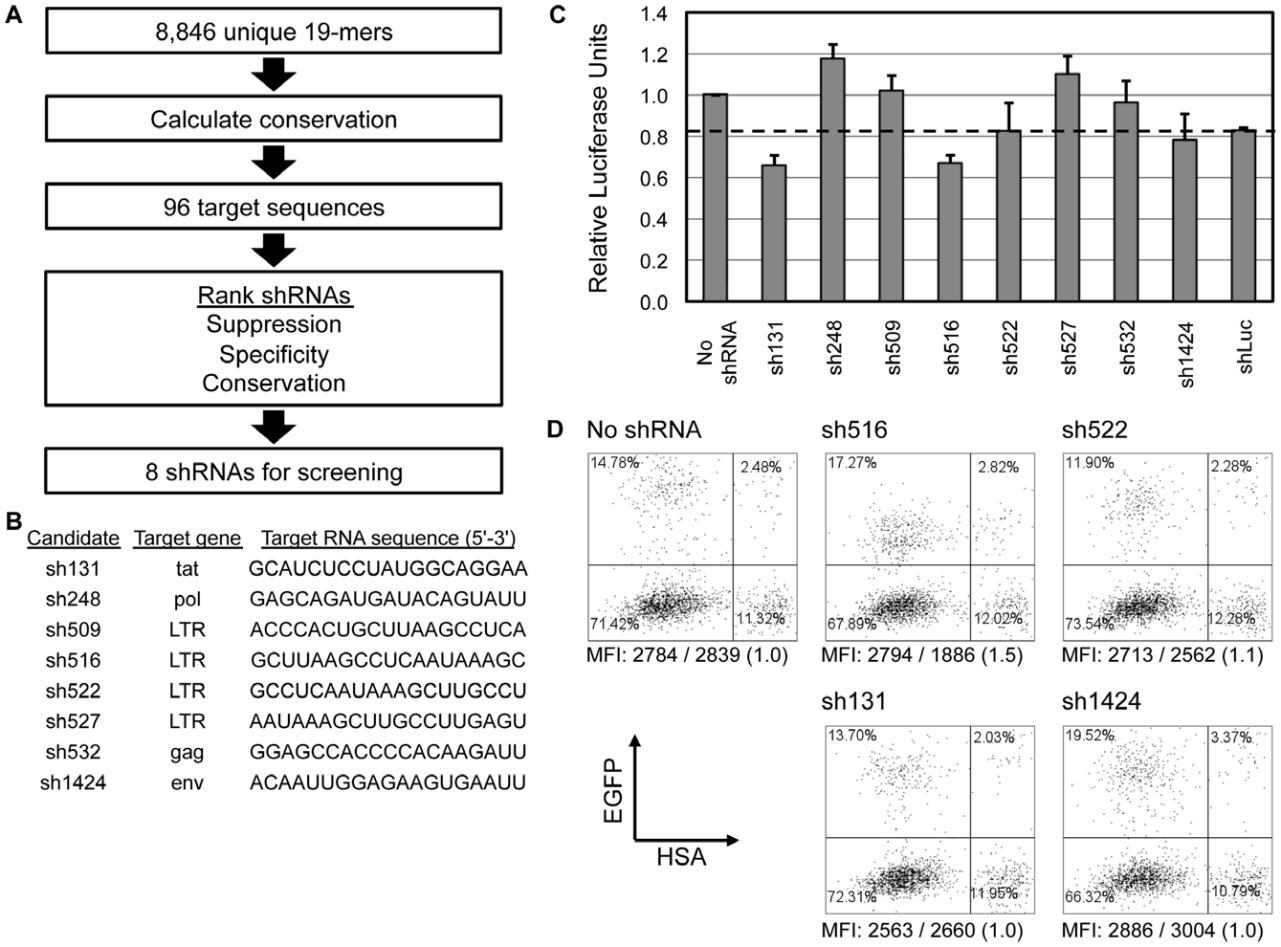
Selection of a single potent anti-HIV-1 shRNA. **A.** Schematic of candidate shRNA screening. McIntyre *et al*. ranked HIV-1_NL4-3_-derived target sequences by percent conservation of published and proprietary HIV-1 sequence databases then ranked the top ninety-six 19-mer target sequences based on suppression, specificity, and percent conservation [40]. The resultant top eight shRNAs were selected for validation in our lentiviral vector based setting. **B.** Candidate shRNA targeted genes and sequences. **C.** HEK-293T cells (7×10^4^) were simultaneously transduced with lentiviral vectors bearing shRNAs at MOI 0.3 and infected with NL4-3.Luc.R-E- at MOI 0.3. Two days post-challenge, cells were harvested and luciferase activities were measured. Luciferase units were normalized by transduction efficiency and protein concentration and made relative to that of the No shRNA control. Relative luciferase units at or below the dashed line (shLuc positive control) were considered sufficient knockdown of luciferase expression. Error bars: mean + standard deviation (SD). **D.** Representative EGFP/HSA expression data of virus-challenged vector-transduced HEK-293T cells. HEK-293T cells (7×10^4^) were transduced with lentiviral vectors bearing anti-HIV-1 shRNAs at MOI 0.3 and then challenged two days post-transduction with NL4-3.HSA.R+.E- at MOI 0.5. Two days post-challenge, cells were harvested and expression of HSA at the cell surface and EGFP was analyzed by flow cytometry. HSA mean fluorescent intensity (MFI) information is shown below plots as “MFI: MFI_EGFP-HSA+_/MFI_EGFP+HSA+_ (MFI_EGFP-HSA+_ ÷ MFI_EGFP+HSA+_ normalized to that of No shRNA cells)”.

We sought identification of shRNAs capable of potent inhibition of HIV-1. Previously published studies had compiled 8,846 HIV-1_NL4-3_-specific 19-mer sequences and ranked 96 target sequences based on their degree of conservation, specificity, and suppressive activities *in vitro* [40]. Based on this screening, we selected eight candidates for additional screening within our lentiviral vector gene therapy setting (Figure 1A). The candidates targeted sequences residing within Gag, Env, Pol, Tat, and LTR, specifically the R-region (Figure 1B). shRNA expression cassettes driven by the human H1 RNA Pol III promoter were cloned into an FG12 lentiviral construct that also expresses EGFP to allow monitoring of vector-transduced cells. The H1 promoter was specifically chosen for its lower level of shRNA expression in order to reduce cytotoxic effects [23]. This selection was based upon our previous experience with sh1005 directed against CCR5 where higher levels of expression driven by the U6 promoter resulted in cytotoxicity [27]. Two rounds of selection were performed using HEK-293T cell lines, lentiviral vectors bearing the shRNA candidates, and VSV-G-pseudotyped HIV-1_NL4-3_ reporter viruses that express either firefly luciferase or murine heat stable antigen (HSA) as a marker gene (NL4-3.Luc.R-E- and NL4-3.HSA.R+.E-, respectively [41], [42]). Efficiencies of shRNA vector transductions were limited to 16–25% to minimize the contribution of multiple vector integrations towards viral inhibition. Based on suppression of gene expression of reporter genes firefly luciferase (Figure 1C) and murine HSA (Figure 1D), we found sh516 to be the most potent candidate.

Importantly, the sh516 target sequence resides within the R-region of the HIV-1 LTR, specifically at the poly(A) hairpin which contains the polyadenylation hexamer motif. As both the 5′ and 3′ LTRs of HIV-1 possess this region, all HIV-1 transcripts, including all spliced transcripts [43], contain two sh516 target sequences. Therefore, we hypothesized that sh516 is capable of down-regulating multiple HIV-1 transcripts, resulting in decreased viral replication to a degree potentially higher than suggested by reporter gene suppression shown in Figure 1. Also, the sh516 target sequence is highly conserved in 88.5% (1786/2019) of all and 95.9% (1002/1046) of clade B HIV-1 sequences found in the Los Alamos National Lab HIV Sequence Database (http://www.hiv.lanl.gov). Based on suppressive activities, potential to down-regulate multiple HIV-1 genes, and high conservation among known HIV-1 strains, we considered sh516 a candidate for anti-HIV-1 combination therapy.

### Concurrent Inhibition of CCR5 and HIV-1 by Combination Vector

**Figure 2:**
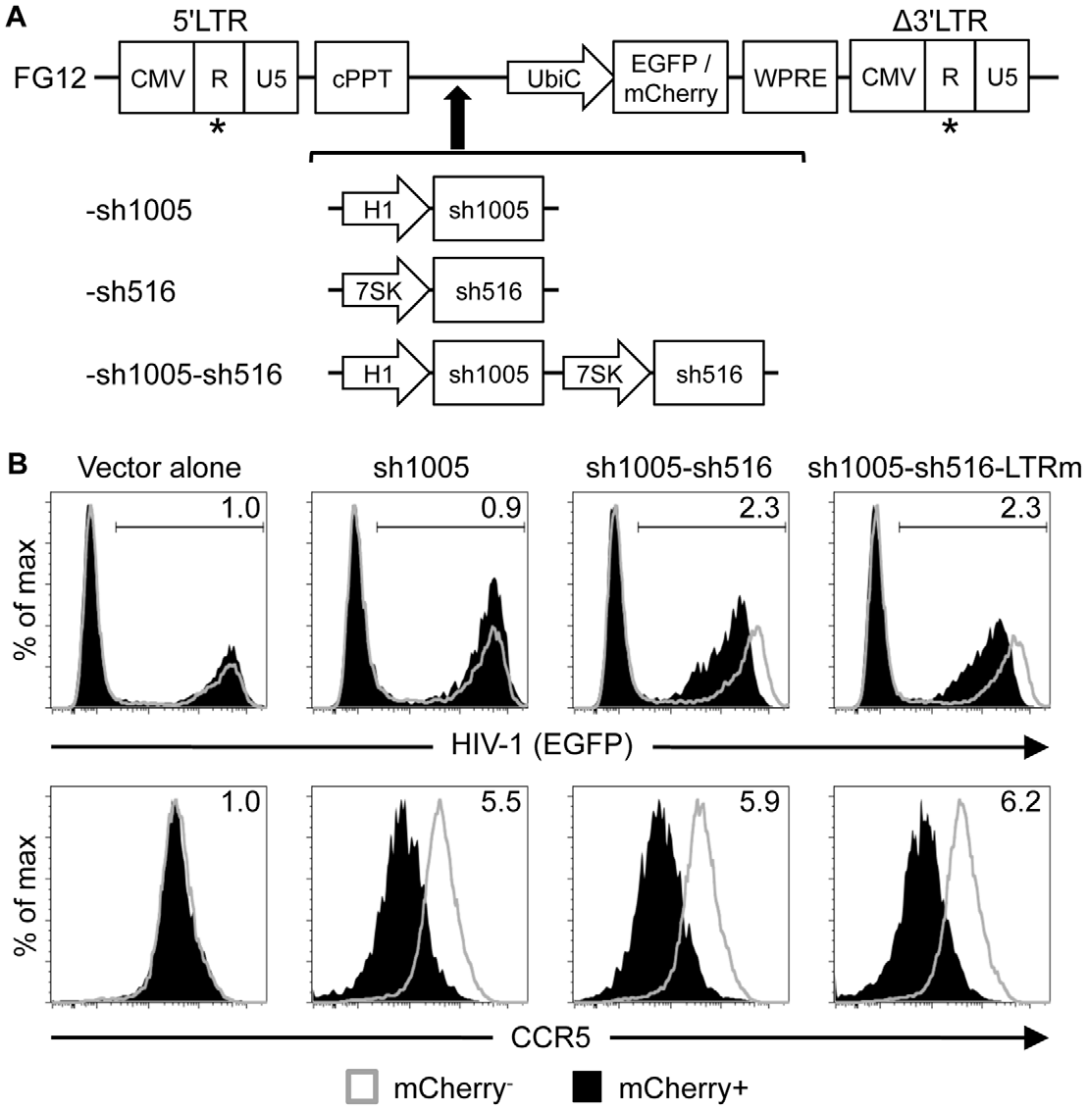
Concurrent down-regulation of CCR5 and HIV-1 via sh1005/sh516 co-expression. **A.** Schematics of lentiviral vectors. Asterisks depict sites of vector LTR mutagenesis in sh516-expressing vectors. **B.** Down-regulation of CCR5 and HIV-1 reporter gene expression. CEM-NKR.CCR5 cells (1×10^5^) were transduced with mCherry-marked lentiviral vectors at MOI 0.5 then seven days later infected with NL4-3 EGFP R-E-at MOI 0.8. EGFP (top panel) and CCR5 (bottom panel) expression was analyzed at three days post-infection. Fold inhibition of gene expression, shown in the top right corner of each histogram, was calculated as MFI in untransduced (mCherry^−^) cells divided by MFI in transduced (mCherry^+^) cells. LTRm: Vector LTR-modified.

Next, we generated a lentiviral vector capable of co-expressing shRNAs targeting CCR5 and HIV-1 to inhibit replication of both R5- and X4- tropic HIV-1. We applied a tandem shRNA expression cassette architecture to our existing CCR5 shRNA-expressing FG12-based lentiviral vector construct (FG12-sh1005) used in previous gene therapy studies [21], [27], [36] to achieve expression of both shRNAs (Figure 2A). For sh516 expression, we used a different Pol III promoter, the human 7SK promoter, to avoid homologous recombination-mediated deletion of vector sequences during reverse transcription [20], [44]. This promoter drives shRNA expression levels similar to that of the H1 promoter, thereby limiting potential cytotoxic effects from shRNA overexpression [20], [45].

To assess the suppressive activities of our resultant combination vector, we challenged vector-transduced CCR5-expressing CEM cells (CEM-NKR.CCR5) [46] with EGFP-marked HIV-1_NL4-3_ reporter virus pseudotyped with G glycoprotein of the vesicular stomatitis virus (VSVG) (NL4-3 EGFP R-E-) [47]. Cells co-expressing sh1005 and sh516 (sh1005-sh516) exhibited HIV-1 marker gene expression levels 2.3 fold lower than those of untransduced cells while control (Vector alone) and solely sh1005-expressing (sh1005) cells expectedly showed no major effect (Figure 2B). Also, CCR5 expression in cells co-expressing sh1005 and sh516 was down-regulated to levels similar to that of solely sh1005-expressing cells (5.9 fold and 5.5 fold, respectively, relative to vector alone). Therefore, our combination vector successfully suppressed expression of CCR5 and HIV-1 reporter gene and sh1005-mediated CCR5 reduction was unaffected by concurrent sh516 expression.

Lentivirally expressed shRNAs might recognize target sequences residing within packaging or transgene-expressing vector constructs, resulting in RNAi-mediated down-regulation of lentiviral genes or delivered transgenes [48] (Figure S1A). To address this concern, we introduced point mutations at the center of the sh516-targeted sequence residing in the vector LTRs of sh516-expressing vectors (Figure S1B) to disrupt recognition by sh516. We selected a U-to-G vector LTR modification based on its improved vector titer and marker gene expression. Vector LTR mutations increased vector titers (Figure S1C) and marker gene expression (Figure S1D) to levels similar to those observed with the sh1005 vector while preserving sh1005 and sh516 activities (Figure 2B). Also, sh1005 expression had no major effect on vector titer or marker gene expression (Figure S1C and S1D). The resultant optimized vector will now be referred to as “Dual sh1005/sh516” and sh1005- and sh516 -expressing vectors as “Mono sh1005” and “Mono sh516,” respectively.

### Inhibition of HIV-1 Replication by Lentiviral Vectors Expressing sh1005, sh516, or Both

**Figure 3:**
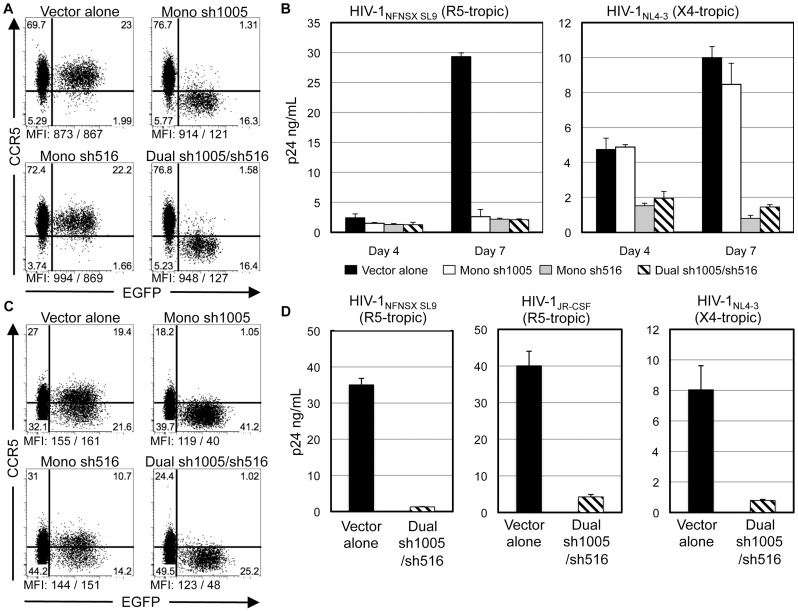
Down-regulation of CCR5 and inhibition of HIV-1 replication in MOLT4-CCR5 cells and PBMCs by Dual sh1005/sh516. **A.** MOLT4/CCR5 (1×10^5^) cells were transduced with lentiviral vectors at MOI 0.5. EGFP and CCR5 expression was assessed three days post-transduction. CCR5 MFIs shown below plots as “MFI: MFI_EGFP−_/MFI_EGFP+_.” Representative data of three independent experiments. **B.** Sorted EGFP^+^ cells (2×10^5^) were infected with either HIV-1_NL4-3_ at MOI 0.5 and HIV-1_NFNSX SL9_ at MOI 5.0. Levels of p24 antigen in culture supernatants were measured by ELISA four and seven days post-infection. Errors bars: mean + SD. **C.** IL-2/PHA stimulated PBMCs (4×10^5^) were transduced with lentiviral vectors at MOI 0.6–1.0. EGFP and CCR5 expression was measured at seven days post-transduction. CCR5 MFIs shown as in **A**. Representative data showing CCR5 expression in vector-transduced PBMCs. **D.** Sorted EGFP^+^ cells (5×10^4^) were infected with either HIV-1_NFNSX SL9_ at MOI 5.0, HIV-1_JR-CSF_ at MOI 1.0, or HIV-1_NL4-3_ at MOI 0.1. p24 production was measured as in **B**.

We next assessed if sh1005/sh516 co-expression can inhibit replication of both X4- and R5- tropic HIV-1. As assessment of viral suppression by marker gene expression is limited to the down-regulation of a single gene product, monitoring p24 production by enzyme-linked immunosorbent assay (ELISA) allows for a more practical evaluation of viral inhibition. We performed viral challenge experiments using replication competent X4-tropic HIV-1_NL4-3_ [49] and HIV-1_NFNSX SL9_ [50], an R5-tropic variant of HIV-1_NL4-3_, and the HIV-1-permissive human CCR5-expressing MOLT4/CCR5 T-cell line [51]. Concurring with CEM.NKR-CCR5 transduction studies, efficient CCR5 down-regulation was observed in cells transduced with Mono sh1005 and Dual sh1005/sh516 vectors, but not with Vector alone or Mono sh516 vectors (Figure 3A). Next, gene-marked cells were sorted by EGFP expression and challenged with either HIV-1_NFNSX SL9_ or HIV-1_NL4-3_ and virus replication was measured by HIV-1 p24 antigen ELISA of culture supernatants. Expression of sh1005 and sh516 individually (Mono sh1005 and Mono sh516) or combined (Dual sh1005/sh516) inhibited replication of HIV-1_NFNSX SL9_ 11.2–14.0 fold relative to vector alone. As expected, Mono sh1005 transduction rendered no significant effect on replication of HIV-1_NL4-3_, whereas sh516 expression inhibited replication by 12.5 and 6.9 fold relative to Vector alone in Mono sh516- and Dual sh1005/sh516- transduced cells, respectively (Figure 3B). These results demonstrated that sh1005 and sh516 possess similar inhibition efficacies against R5-tropic HIV-1 replication and that expression of sh516 alone as well as sh516/sh1005 co-expression can inhibit replication of both X4- and R5- tropic HIV-1.

We then determined if Dual sh1005/sh516 can down-regulate CCR5 expression and inhibit X4- and R5- tropic HIV-1 replication in primary human peripheral blood mononuclear cells (PBMCs). shRNA quantitation via real-time quantitative reverse transcription PCR (qRT-PCR) showed that sh516 expression did not have significant effects on sh1005 expression in Dual sh1005/sh516-transduced cells (Table S1). Mono sh1005 and Dual sh1005/sh516 had similar levels of CCR5 down-regulation (13.0 and 8.8 fold, respectively, by percentages of CCR5^+^ cells in untransduced and transduced populations) while no effect on CCR5 expression was observed in cells transduced with either Vector alone or Mono sh516 vectors (Figure 3C). FACS-sorted EGFP^+^ cells were subjected to viral challenge with X4-tropic HIV-1_NL4-3_ and R5-tropic strains HIV-1_NFNSX SL9_ and HIV-1_JR-CSF_, a clinical isolate [52]. Dual sh1005/sh516 inhibited replication of HIV-1_NFNSX SL9_, HIV-1_JR-CSF_ and HIV-1_NL4-3_ by, 27.2, 9.4, and 10.2 fold, respectively, relative to that with Vector alone (Figure 3D). These results demonstrated that sh1005/sh516 co-expression efficiently down-regulated CCR5 expression and inhibited both X4- and R5- tropic HIV-1 replication in PBMCs *in vitro*.

### Stable sh1005/sh516 Co-Expression with Minimal Effects on Cell Viability and HSPC Differentiation

**Figure 4:**
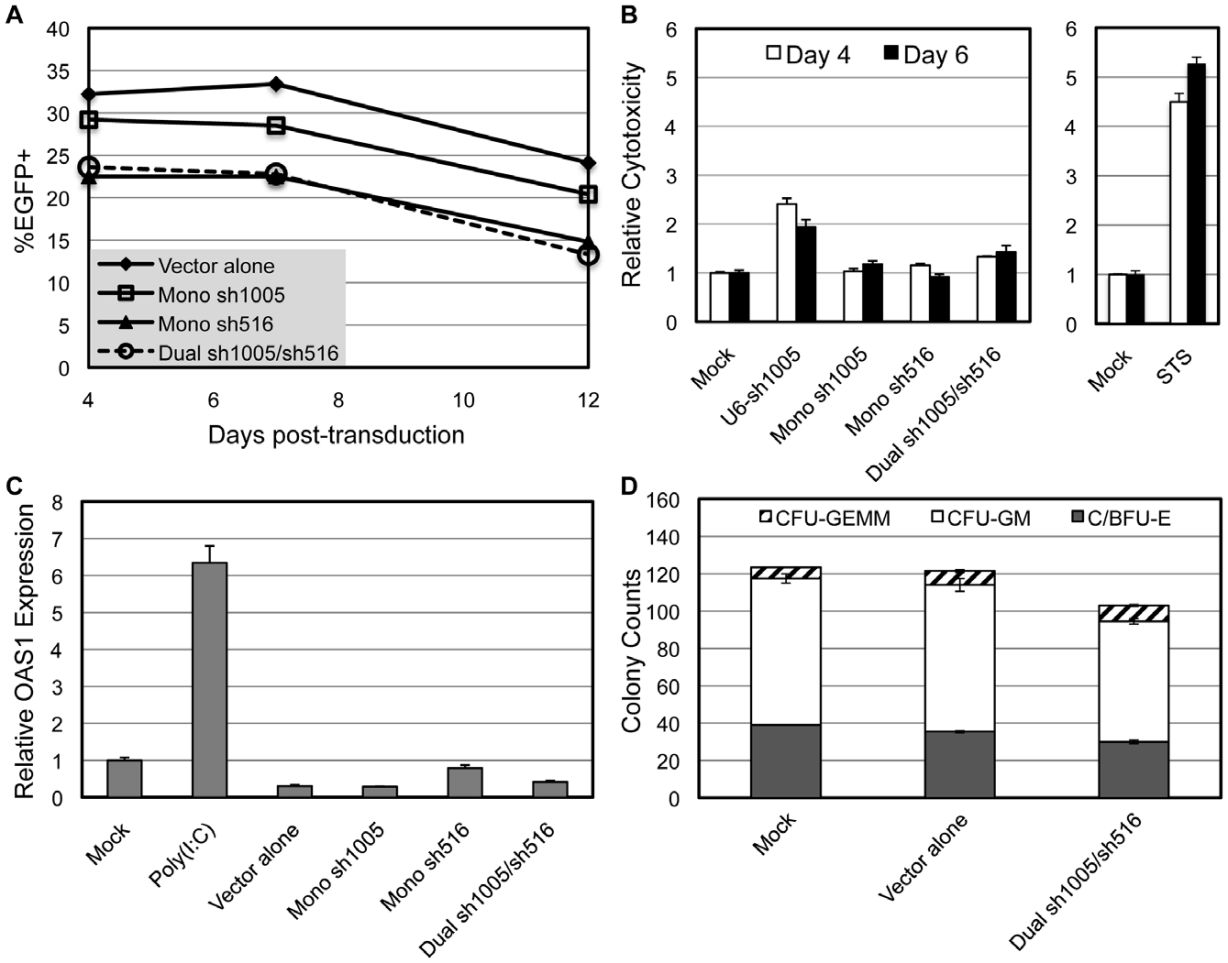
Vector stability and the effects of sh1005/sh516 co-expression on cell viability and HSPC differentiation potential. IL-2/PHA-stimulated PBMCs (4×10^5^) were transduced with lentiviral vectors at MOI 0.6–1.0. **A.** Representative data showing EGFP expression in vector-transduced PBMCs over time. **B.** Vector-transduced cells were subjected to CytoTox-Glo^TM^ Cytotoxicity Assay (Promega) four and six days post-transduction. Relative cytotoxicities were calculated by dividing the dead cell count by the total cell count and normalizing to that of mock-transduced cells. 1 μM Staurosporine (STS) and U6 promoter-driven sh1005 expression (U6-sh1005) served as positive controls. Error bars: mean + SD. **C.** OAS1 mRNA expression relative to β-actin was assessed by qRT-PCR analysis of total RNA isolated from vector-transduced PBMCs. PBMCs harvested two days post-electroporation with 500 pg/μL poly(I:C) served as positive control. Values were normalized to those of mock-transduced cells. Error bars: mean + SD. **D.** Cytokine-pre-stimulated mPB-CD34^+^ cells were transduced with lentiviral vectors at MOI 10. HSPC differentiation potential was assessed by counting colony forming units produced from mock- and vector- transduced mPB-CD34^+^ cells plated on semi-solid methylcellulose plates one day after transduction. CFU-GEMM: granulocyte/erythrocyte/macrophage/megakaryocyte colony forming units. C/BFU-E: erythroid colony/burst forming units. CFU-GM: granulocyte/monocyte colony forming units. Error bars: range of values between duplicate samples. Representative data of three independent experiments.

We examined the persistence of the integrated vector in Dual sh1005/sh516-transduced MOLT4/CCR5 cells as well as PBMCs. We found previously that shRNA expression driven by the U6 promoter in PBMCs resulted in a rapid decline in transduced cells over time [23]. However, stable EGFP expression and CCR5 down-regulation was maintained in Dual sh1005/sh516-transduced MOLT4/CCR5 for over 2 months (data not shown). Also, vector-transduced PBMCs exhibited marker gene expression profiles nearly identical to that of Vector alone-transduced cells (Figure 4A). These results demonstrated sustained vector stability in Dual sh1005/sh516-transduced cells.

We next determined if sh1005/sh516 co-expression had adverse effects on cell viability using PBMCs. We and other groups have shown that high levels of shRNA expression can decrease cell viability [23], [53], [54] and may activate the interferon (IFN) response pathway which can cause attenuated cell growth and apoptosis [54]. We have previously shown that U6 promoter-driven sh1005 expression, which is six times higher than that with the H1 promoter, may diminish cell viability of vector-transduced PBMCs [27]. To monitor acute cytoxicity, we subjected vector-transduced PBMCs to the CytoTox-Glo^TM^ Cytotoxicity Assay (Promega). Expression of sh1005, sh516, or both concurrently had no major effect on the dead:total cell number ratio (Figure 4B). To determine if sh1005/sh516 co-expression induced an IFN response, we assessed the expression of 2′-5′-oligoadenylate synthetase 1 (OAS1), a widely-used indicator of IFN induction [54], in vector-transduced PBMCs. OAS1 qRT-PCR analysis did not detect significant OAS1 up-regulation in PBMCs expressing either sh1005, sh516, or both concurrently while electroporation with IFN response-inducing polyinosinic-polycytidylic acid [poly(I:C)] induced expression of OAS1 6.3 fold relative to mock-treated cells (Figure 4C). Therefore, we concluded that sh516 expression alone or with sh1005 had no obvious cytotoxicity and did not activate the IFN response pathway.

As our HSPC-based clinical approach relies heavily on the capability of gene-modified mobilized peripheral blood CD34^+^ (mPB-CD34^+^) cells to differentiate into the various hematopoietic lineages, we next determined if sh1005/sh516 co-expression had adverse effects on HSPC differentiation potential. Vector-transduced mPB-CD34^+^ cells were subjected to a semi-solid methylcellulose-based colony forming cell assay [28]. Relative to Vector alone-transduced cells, sh1005/sh516 co-expression had no adverse effects on colony forming units for granulocyte/erythrocyte/macrophage/megakaryocyte, erythroid, and granulocyte/monocyte, suggesting that Dual sh1005/sh516-transduced HSPCs maintained hematopoietic differentiation potential *in vitro* (Figure 4D). Altogether, these data demonstrated that co-expression of sh1005 and sh516 was stable and had no obvious adverse effects on cell viability or HSPC multi-lineage differentiation *in vitro*.

### Reconstitution and CCR5 Down-Regulation by Dual sh1005/sh516-Transduced HSPC in hu-BLT Mice

The hu-BLT mouse model allows assessment of engraftment and reconstitutive properties of human HSPCs *in vivo* [37], [38]. Systemic reconstitution in this model requires myeloablation, via total body irradiation or administration of drugs such as Busulfan [55], of recipient immunocompromised mice followed by transplantation of a human fetal thymus/liver (thy/liv) implant as well as intravenous (IV) injection of FL-CD34^+^ cells. We previously demonstrated in the hu-BLT mouse model successful engraftment and differentiation of transplanted sh1005 vector-transduced human HSPCs and resultant down-regulation of CCR5 expression in T-cells and monocytes/macrophages in peripheral blood and systemic lymphoid organs, including GALT [36]. We extended our hu-BLT mouse model to assess engraftment, repopulation, and anti-viral capacities of Dual sh1005/sh516-transduced HSPC.

**Figure 5:**
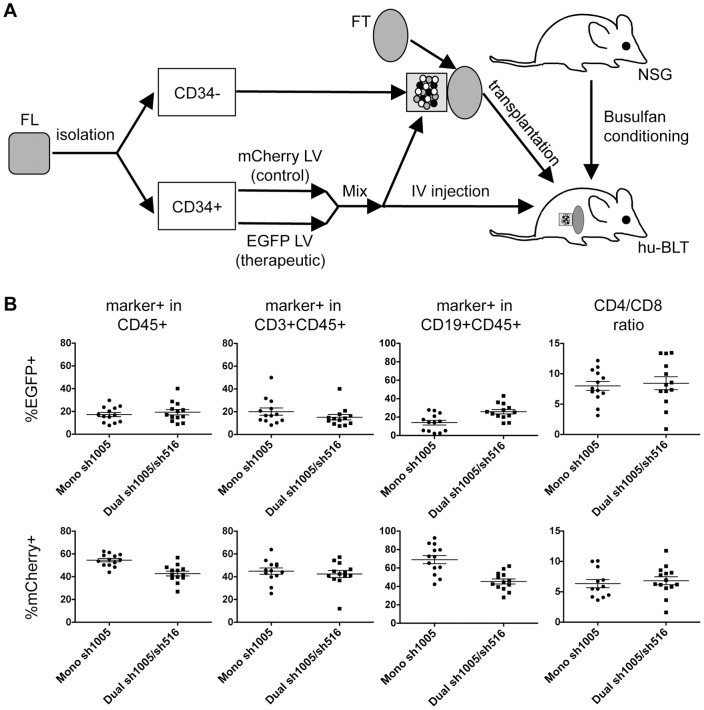
Reconstitution of Dual sh1005/sh516-transduced HSPCs in humanized BLT mice. **A.** Schematic of generating vector-transduced HSPC-transplanted hu-BLT mouse. NSG mouse is treated with Busulfan 24 hours pre-transplantation. CD34^+^ and CD34^−^ cells are isolated from human fetal liver (FL). CD34^+^ cells are transduced with either therapeutic (EGFP-marked) or control (mCherry-marked) vectors. Therapeutic vector- and control vector- transduced CD34^+^ cells are mixed at a 50 50 ratio. The cell mixture is then 1) combined with CD34^−^ cells, solidified with Matrigel, and implanted under the kidney capsule with a human fetal thymus segment (FT) and also 2) intravenously injected. **B.** EGFP and mCherry reporter gene expression was monitored in human CD45^+^, CD3^+^CD45^+^, and CD19^+^CD45^+^ cells within a gated lymphocyte population in peripheral blood at twelve weeks post-transplantation. CD4/CD8 ratios were analyzed in mCherry- and EGFP- marked CD3^+^CD45^+^ cells. Data were generated from n = 13 mice for both Mono sh1005- and Dual sh1005/sh516- HSPC-transplanted animals from an aggregate of three donors. Error bars: mean + standard error of mean (SEM) in n = 13 per group.

As previously described [36], we used a two-fluorescent reporter system to simultaneously monitor Dual sh1005/sh516- (EGFP^+^) and control vector- (mCherry^+^) transduced cells, allowing comparative assessment of stability, engraftment, and specificity of CCR5 reduction within the same animal. We transplanted NOD.Cg-*Prkdc^scid^ Il2rg^tm1Wjl^*/SzJ (NSG) mice with either Mono sh1005- or Dual sh1005/sh516- transduced HSPC to allow side-by-side evaluation of therapeutic efficacies of sh1005/sh516 co-expression and previously demonstrated sh1005 expression. FL-CD34^+^ cells isolated from three independent donors were transduced with mCherry-marked control vector, EGFP-marked Mono sh1005, or EGFP-marked Dual sh1005/sh516 with a multiplicity of infection (MOI) of three. Mean vector transduction efficiencies in FL-CD34^+^ cells used for transplantation were 79.1%, 69.4%, and 63.4% for Mono sh1005, Dual sh1005/sh516, and control vectors, respectively. The increased transduction efficiencies were used to improve *in vivo* repopulation of marked cells. Busulfan-conditioned NSG mice received a 50∶50 mixture of control vector- and either Mono sh1005- or Dual sh1005/sh516- transduced FL-CD34^+^ cells transplanted with Matrigel under the kidney capsule with a thymus segment as well as IV injection of transduced FL-CD34^+^ cells (Figure 5A).

We assessed human cell engraftment in peripheral blood of transplanted mice for up to twelve weeks post-transplantation by flow cytometry analysis as previously described [36]. Human CD45^+^ (all-lymphocyte marker) cells were detected in peripheral blood isolated from all Mono sh1005- (7.3–69.2% of total cells, n = 13) and Dual sh1005/sh516- (20.4–80.6% of total cells, n = 13) transplanted mice. Within a lymphocyte-gated population, proportions of CD3^+^CD45^+^ (T-lymphocyte marker, ∼51.4%±24.5% with Mono sh1005 and ∼54.7%±28.1% with Dual sh1005/sh516) and CD19^+^CD45^+^ (B-lymphocyte marker, ∼40.5%±22.8% with Mono sh1005 and 38.2%±26.0% with Dual sh1005/sh516) cells were similar between Mono sh1005- and Dual sh1005/sh516- transplanted mice, suggesting sh516 expression does not skew repopulation of engineered HSPC. Reconstitution of hematopoietic lineages from Dual sh1005/sh516-transduced HSPCs was observed in all transplanted mice, yielding marking in lymphocyte (8.9–40.1% EGFP^+^), T-cell (7.5–40.1% EGFP^+^) and B-cell (13.4–43.0% EGFP^+^) populations within a lymphocyte-gated population (Figure 5B). Observed levels of reconstitution of EGFP-marked lineages may be underestimations due to inclusion of control vector-transduced HSPCs and lack of selective pressure supporting EGFP-marked cells (*e.g*. HIV-1 infection). Comparable levels of EGFP and mCherry marking in CD45^+^, CD3^+^CD45^+^, and CD19^+^CD45^+^ populations within a lymphocyte-gated population as well as similar CD4/CD8 ratios were detected between Mono sh1005- and Dual sh1005/sh516- transplanted mice (Figure 5B). We next examined in a mouse transplanted with Dual sh1005/sh516-transduced HSPCs marker gene expression in human CD45^+^ cells in systemic lymphoid organs eleven weeks post-transplantation. Flow cytometry analysis revealed EGFP marking in CD45^+^ cells derived from peripheral blood, bone marrow, spleen, and GALT (Table 1). Altogether, these data demonstrated that transplantation of Dual sh1005/sh516-transduced HSPCs could successfully engraft and differentiate into human T-cells in peripheral blood and systemic lymphoid organs with engraftment and reconstitution efficiencies similar to those with Mono sh1005-transduced HSPCs.

**Table 1:** Human hematopoietic differentiation of Dual sh1005/sh516-transduced HSPC in multiple lymphoid organs.^a^

	CD45+	CD3+^b^	CD4+^c^	CD8+^c^	CD19+^b^	CD14+^b^
**%EGFP^+^**						
Blood	15.8	12.3	11.2	19.3	13.8	13.9
Bone marrow	13.2	8.0	7.0	14.3	13.6	22.9
Spleen	17.3	13.0	11.9	18.1	15.8	13.4
GALT	17.6	10.0	8.2	20.4	28.1	11.8
**%mCherry^+^**						
Blood	54.3	42.2	39.4	28.0	64.3	65.3
Bone marrow	57.7	45.3	43.2	30.1	57.8	55.5
Spleen	55.9	42.2	39.8	31.6	61.5	18.5
GALT	50.7	38.8	35.9	23.7	54.7	4.3

a Reporter gene marking in tissues isolated eleven weeks after transplantation.

b In CD45^+^ population.

c In CD45^+^CD3^+^ population.

**Figure 6:**
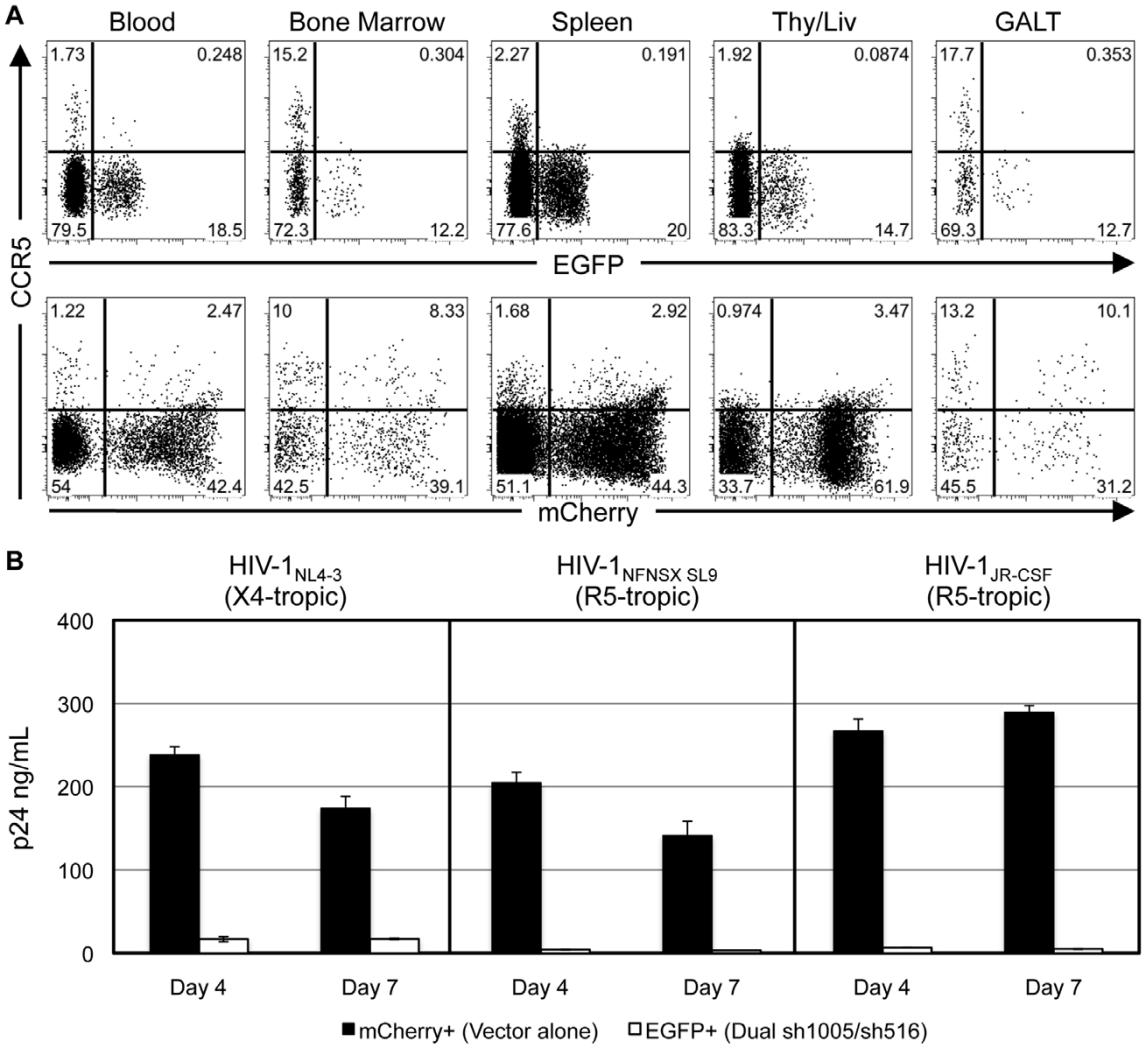
Down-regulation of CCR5 in systemic lymphoid tissues and inhibition of HIV-1 replication *ex vivo* by Dual sh1005/sh516. Tissues were isolated from a Dual sh1005/sh516-transduced HSPC-transplanted mouse at eleven weeks post-transplantation. **A.** Representative data showing CCR5 expression in EGFP- and mCherry- marked human CD4^+^CD3^+^CD45^+^CD19^−^ T-cells within a gated lymphocyte population. **B.** Splenocytes were depleted of human CD8^+^ cells and murine CD45^+^ cells and then stimulated with PHA/IL-2 for two days. Five days later, EGFP^+^ and mCherry^+^ cells were then sorted by FACS at >97% purities. Sorted cells (5×10^4^) were then infected with either HIV-1_NL4-3_ at MOI 0.5, HIV-1_NFNSX SL9_ at MOI 5.0, or HIV-1_JR-CSF_ at MOI 1.0 for four hours. Cells were then washed five times before culturing. HIV-1 replication was monitored by p24 ELISA analysis of culture supernatants at four and seven days post-infection. Error bars: mean + SD. Representative data of three independent experiments.

We assessed siRNA expression and CCR5 down-regulation in CD4^+^ T-cells in peripheral blood and systemic lymphoid organs from the previously described transplanted mouse. Similar levels of vector integration and expression of si1005 and si516 were observed among lymphocytes derived from bone marrow and spleen (Table S1). CCR5 expression was efficiently reduced by 1.6–7.5 fold in all tissues analyzed, including bone marrow (7.1 fold) and GALT (7.5 fold) where CCR5 is expressed at high levels (Figure 6A). Despite decreased levels of siRNA expression relative to those seen *in vitro* (Table S1), observed levels of CCR5 down-regulation with Dual sh1005/sh516 were similar to those seen with our previous hu-BLT studies using HSPC's transduced with Mono sh1005 [36]. These results demonstrated that sh1005 expressed concurrently with sh516 successfully down-regulated CCR5 expression in CD4^+^ T-cells in multiple systemic lymphoid tissues *in vivo*.

### R5- and X4- Tropic HIV-1 is Inhibited in *Ex Vivo* Isolated sh1005/sh516 Co-Expressing Splenocytes

We have previously shown that CCR5-down-regulated cells isolated from a hu-BLT mouse transplanted with Mono sh1005-transduced HSPCs were resistant to R5-tropic, but not X4-tropic, HIV-1 infection *ex vivo* [36]. We examined the susceptibility of cells isolated from a hu-BLT mouse transplanted with Dual sh1005/sh516-transduced HSPC to X4- as well as R5- tropic HIV-1 infection. Sorted spleen-derived EGFP^+^ and mCherry^+^ human T-cells were challenged with either HIV-1_NL4-3_, HIV-1_NFNSX SL9_, or HIV-1_JR-CSF_. As expected, mCherry^+^ cells were highly susceptible to infection by all three HIV-1 strains. In contrast, viral replication in R5-tropic HIV-1-infected EGFP^+^ was inhibited 40–56 fold relative to that with mCherry^+^ cells. Unlike with previously published studies with Mono sh1005 [36], sh1005/sh516 co-expression inhibited X4-tropic viral replication 10–14 fold relative to that with mCherry^+^ cells (Figure 6B). These results demonstrated that sh1005/sh516 co-expression efficiently inhibited both X4- and R5- tropic HIV-1 replication in spleen-derived T-cells *ex vivo*.

### Both R5- and X4- Tropic HIV-1-Mediated CD4^+^ T-Cell Depletion are Inhibited by sh1005/sh516 Co-Expression in hu-BLT Mice

**Figure 7:**
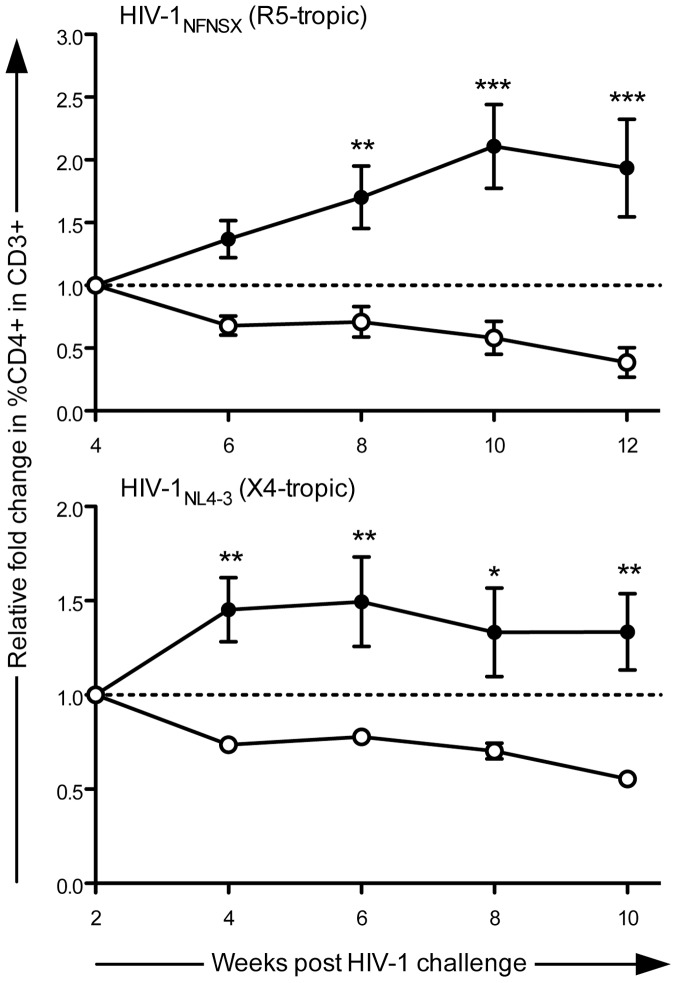
Protection from R5- and X4- tropic HIV-1-mediated CD4^+^ T-cell loss in hu-BLT mice by Dual sh1005/sh516. hu-BLT mice transplanted with Dual sh1005/sh516-transduced HSPC were challenged intravenously with either HIV-1_NFNSX_ or HIV-1_NL4-3_ ∼12 weeks post-transplantation. Levels of marked CD4^+^ T-cells, shown as percentages within total CD3^+^ cells in peripheral blood (shown in Table S2), were normalized to that of the time point preceding decline of percentages of control-vector transduced cells to determine relative fold change over time. Values above and below 1.0 on the y-axis depict increases or decreases, respectively, in CD4^+^ T-cell levels. Closed circles: EGFP-marked. Open circles: mCherry-marked. Error bars: mean + SEM in n = 5 and n = 4 for HIV-1_NFNSX_- and HIV-1_NL4-3_- infected mice, respectively. Statistical significance calculated by two-way ANOVA/Bonferroni post-test. *  =  p<0.05, **  =  p<0.01, ***  =  p<0.001.

To assess if Dual sh1005/sh516-transduced HSPC-derived CD4^+^ T-cells are resistant against HIV-1-mediated cell depletion *in vivo*, we intravenously administered transplanted hu-BLT mice with either HIV-1_NL4-3_ (n = 4) or HIV-1_NFNSX_ [56] (n = 5) then monitored levels of EGFP- and mCherry- marked CD4^+^ T-cells as percentages within total peripheral blood CD3^+^ cells for 10–12 weeks. Levels of control vector-transduced CD4^+^ T-cells began to decline after week 4 and week 2 post-viral challenge with HIV-1_NFNSX_ and HIV-1_NL4-3_, respectively. Therefore, comparisons of CD4^+^ T-cell levels were assessed 4–12 weeks post-HIV-1_NFNSX_ infection and 2–10 weeks post-HIV-1_NL4-3_ infection. As mCherry^+^ CD4^+^ T-cell levels declined in all infected mice (n = 9) over time (−19 to −92% with R5-tropic HIV-1 and −37 to −53% with X4-tropic HIV-1), EGFP^+^ CD4^+^ T-cell levels increased in five out of five HIV-1_NFNSX_-infected (16 to 217%) and three out of four HIV-1_NL4-3_-infected (16 to 92%) hu-BLT mice. However with the remaining HIV-1_NL4-3_-infected mouse, the decrease in EGFP^+^ CD4^+^ T-cell levels (−1%) was less than observed with mCherry^+^ CD4^+^ T-cells (−41%) (Table S2), suggesting slower T-cell depletion in EGFP^+^ cells. Due to observed differences in reconstitution levels among the mice, CD4^+^ T-cell levels were normalized to that of the time point preceding HIV-1-mediated decline of control vector-transduced CD4^+^ T-cell levels. Following HIV-1 infection with either X4- or R5- tropic viral strains, average relative fold change in CD4^+^ T-cell levels increased with EGFP^+^ cells (up to ∼1.5 and ∼2.1, respectively), but decreased with mCherry^+^ cells (up to ∼1.8 and ∼2.6, respectively), with statistically significant differences in relative fold changes between EGFP- and mCherry- marked CD4^+^ T-cell levels appearing as early as week 8 post-R5-tropic HIV-1 infection and week 4 post-X4-tropic HIV-1 infection (Figure 7). These results showed that CD4^+^ T-cells derived from transplanted Dual sh1005/sh516-transduced HSPC resisted depletion by both R5- and X4- tropic HIV-1 infection *in vivo*.

## Discussion

Transplantation of engineered HSPCs holds great promise for stably controlling HIV-1 infection. Unlike current antiretroviral therapies, HSPC-based gene therapy may be potentially curative, reversing the virus's damage inflicted on the immune system, with a single or few administrations. While high viral inhibition can be achieved *in vitro*, recent HSPC-based approaches observed reduced vector titers possibly due to high complexity of vector constructs and/or inclusion of shRNAs which target both vector and HIV-1 genes [57], [58]. Interestingly, a recent clinical trial evaluating an RNA-based combination approach revealed persistence of engineered cells for at least 24 months in patients, but no clinical benefit was observed due to low engraftment [26], illustrating the necessity of combining high reconstitution with potent inhibition. Limited *in vivo* efficacies observed with previous HSPC-based gene therapy strategies may be due to lack of a small animal model system to support HIV-1 replication to aid preclinical assessment. The hu-BLT mouse model affords high T-cell reconstitution efficiencies, human thymus-mediated T-cell development, and engraftment in clinically relevant systemic lymphoid organs such as GALT, a primary site of HIV-1 infection [39]. We previously demonstrated CCR5 downregulation *in vivo* via CCR5 shRNA-transduced HSPC transplantation into hu-BLT mice, thus pioneering the application of the hu-BLT system for AIDS gene therapy development.

To improve anti-viral efficacy and to address potential drawbacks associated with a gene therapy strategy based solely on CCR5 down-regulation, such as viral escape mutagenesis and ineffectiveness against X4-tropic HIV-1 infection, we have incorporated an HIV-1 transcript-targeting shRNA into our current sh1005-expressing vector. Our screening for anti-HIV-1 shRNAs recognizing highly conserved regions and retaining potency at low expression levels revealed a candidate that targets the HIV-1 LTR. Although the sh516 target site is involved in a secondary RNA structure which may dampen RNAi knockdown efficiencies, the high potency of sh516 may be due to the stability of its target site structure (ΔG = −15.3 kcal/mol) [59] which has been shown to be optimal for RNAi efficiency [60]. Directing RNAi toward the sh516 target sequence provides several advantages: 1) high conservation across multiple R5- and X4- tropic strains may provide broad protection; 2) all transcripts derived from HIV-1 genomes bearing the sh516 target sequence within their LTR R regions are susceptible to RNAi, resulting in efficient inhibition of viral replication; and 3) high vector titers (5×10^8^–1×10^9^ transduction units per mL after 200-fold concentration) can be routinely achieved after minor vector LTR modification to a vector LTR region, which is suggested by previous mutagenesis studies of the HIV-1 poly(A) hairpin to tolerate minor changes [59], precluding vector transcript degradation by expressed shRNAs. *In vitro* characterization of sh1005/sh516 co-expression revealed effective inhibition of both R5- and X4- tropic HIV-1 replication with no obvious adverse effects on cell viability or hematopoietic differentiation.

Similar to our *in vivo* CCR5 down-regulation studies, we assessed the engraftment and reconstitution properties of Dual sh1005/sh516-transduced HSPC in the hu-BLT mouse model. We observed successful engraftment of engineered HSPCs in all transplanted animals. Dual sh1005/sh516-transduced HSPC successfully differentiated into hematopoietic lineages in all transplanted mice. Increased levels of reconstitution from Dual sh1005/sh516-transduced HSPC may be expected with exclusion of control vector-transduced HSPCs from the transplant. Marker gene expression and efficient CCR5 down-regulation were observed in Dual sh1005/sh516-transduced HSPC-derived CD4^+^ T-cells isolated from peripheral blood, spleen, bone marrow, and GALT, suggesting potential anti-viral protection in various systemic lymphoid tissues. Furthermore, sh1005/sh516 expression effectively inhibited both R5- and X4-tropic HIV-1 replication in spleen-derived T-cells *ex vivo*. Lastly, Dual sh1005/sh516-transduced HSPC-derived CD4^+^ T-cells resisted depletion by both R5- and X4- tropic HIV-1 infection in 5/5 and 3/4 transplanted hu-BLT mice, respectively. The observed difference in effectiveness against R5- and X4- tropic viruses may be due to sh1005/sh516 co-expression providing two anti-R5 virus reagents (sh1005 and sh516), but only one anti-X4 virus reagent (sh516). To our knowledge, this is the first demonstration of an shRNA-based HSPC gene therapy that confers resistance against HIV-1 strains utilizing either CCR5 or CXCR4 co-receptors to CD4^+^ T-cells in the hu-BLT model.

Our current study demonstrates the potential therapeutic efficacy of our combinatorial shRNA-based gene therapy strategy. Even so, any anti-HIV-1 therapy is potentially susceptible to emergence of therapy-resistant viral strains, necessitating the concurrent use of multiple therapeutic reagents. Co-expression of two therapeutic genes potentially has a lower chance for viral resistance than with a monotherapy. Moreover, shRNA efficacy can be diminished with minor mutagenesis of the targeted region [61], [62]. As such, the high mutational rate of HIV-1 may require additional reagents to minimize chances of viral escape. The small size of the sh1005/sh516 tandem shRNA expression cassette allows future incorporation of supplementary therapeutics such as additional shRNAs or proteins such as fusion inhibitors, host restriction factors, and engineered T-cell receptors (reviewed in [5], [7], [63]), further increasing inhibition efficacy and decreasing the chances of viral resistance. The challenge for HSPC-based gene therapy for long-term control of HIV-1 infection will be to identify the combination of reagents that provides high repopulation, safe sustained maintenance, and effective anti-HIV-1 activity.

## Materials and Methods

### Ethics Statement

Human PBMCs were obtained without identifying information from the UCLA Center for AIDS Research (CFAR) Virology Core Laboratory in accordance with UCLA Institutional Review Board (IRB) approved protocols along with an IRB-approved written consent form. Human fetal tissues were obtained without identifying information from the UCLA CFAR Gene and Cellular Therapy Core Laboratory and Advanced Biosciences Resources (Alameda, CA) under federal and state regulations and their use did not require IRB approval. Animal research described in the study was conducted under UCLA's Chancellor's Animal Research Committee (Institutional Animal Care and Use Committee [IACUC]) Protocol Number 2007–092 in accordance with guidelines for housing and care of laboratory animals of the National Institutes of Health (NIH) and the Association for the Assessment and Accreditation of Laboratory Animal Care (AALAC) International. All efforts were made to minimize pain and discomfort for the animals.

### Lentiviral Vector Construction and Production

For initial shRNA screening, H1-promoter-driven shRNA expression cassettes were PCR-amplified from pBS hH1-3 [27] using M13 forward and shRNA-specific (GR01-GR09) reverse primers and then inserted between the XbaI and XhoI sites of FG12. To generate 7SK-promoter-driven sh516 expression cassette, pUC57 possessing the 7SK promoter followed by multiple restriction enzyme sites was generated by Genescript (Piscataway, NJ). Next, oligos GR10 and GR11 were synthesized, annealed, and cloned downstream of the 7SK promoter. The 7SK-sh516 expression cassette was cloned into FG12 and FG12-sh1005. To generate Mono sh516 and Dual sh1005/sh516, vector LTRs of sh516-expressing FG12 plasmids were mutated using GR12 primer and Quikchange Multi Site-Directed Mutagenesis Kit (Agilent Technologies, Santa Clara, CA) according to manufacturer's instructions. Resulting plasmids were confirmed by restriction enzyme digestion and DNA sequencing. Detailed sequence information can be provided upon request. Sequences of primers used for vector construction can be found in Table S3. VSVG-pseudotyped lentiviral vector stocks were produced by calcium phosphate-mediated transient transfection of HEK-293T cells, as previously described [22]. Vector stocks were titered on HEK-293T cells based on EGFP or mCherry expression.

### Cells and Culture

HEK-293T cells were cultured as previously described [28]. CEM.NKR-CCR5 and MOLT4/CCR5 were obtained from the AIDS Research and Reference Reagent Program of the National Institutes of Health and maintained, respectively, in Iscove's modified Eagle's medium (Sigma-Aldrich, St. Louis, MO) containing 10% FCS, GPS (100 U/ml penicillin, 100 μg/ml streptomycin and 2 mM glutamine) as well as Roswell Park Memorial Institute 1640 medium (Life Technologies, Carlsbad, CA) containing 10% FCS, GPS, and 1 mg/ml Geneticin (Life Technologies). Human primary PBMCs were isolated, stimulated, and cultured as previously described [23]. Prior to infection with replication competent HIV-1, marker-positive cells were isolated by FACS Aria cell sorter (BD Biosciences, San Jose, CA) to >95% purity and PBMCs were CD8-depleted with CD8 microbeads (Miltenyi Biotec, Cologne, Germany) per manufacturer's instructions.

### Lentiviral Vector Transduction

Transductions of HEK-293T, CEM.NKR-CCR5, and MOLT4/CCR5 were performed with indicated cell numbers and multiplicities of infections (MOIs) for 2 hours in the presence of 8 μg/ml of polybrene. Transductions of PBMCs were performed as previously described [23] with indicated cell numbers and MOIs. mPB-CD34^+^ cells (9.8×10^4^), provided by AllCells (Emeryville, CA), were pre-stimulated with SCF, TPO, Flt-3L, and IL-3 (R&D Systems, Minneapolis, MN) at 50 ng/mL each in X-VIVO 10 medium (Lonza, Basel, Switzerland), 1x GlutaMax (Life Technologies), 0.5x Penn-Strep (MP Biomedicals, Solon, Ohio) for 24 hours then transduced with lentiviral vectors at MOI 10 for 21 hours.

### HIV-1 Production and Infection

pNL4-3, pNL4-3.Luc.R-E-, and pNL4-3.HSA.R+.E- were obtained from the AIDS Research and Reference Reagent Program of the National Institutes of Health. HIV-1_NL4-3_, HIV-1_NFNSX_, HIV-1_NFNSX SL9_, and HIV-1_JRCSF_ as well as VSVG-pseudotyped reporter viruses NL4-3.Luc.R-E-, NL4-3.HSA.R+.E-, and NL4-3 EGFP R-E- were produced by calcium phosphate transfection of HEK-293T cells as previously described [36]. Viral supernatants were passed through 0.22 μm filters and stored at −80°C. Virus stocks were titered on Ghost(3)X4/R5 [64] cells based on EGFP expression. Viral infections were performed with indicated cell numbers and MOIs for 4 hours in the presence of 8 μg/ml polybrene. HIV-1 p24 ELISA analyses of supernatants of infected cells were performed in triplicate.

### Generation of hu-BLT Mice

Isolation of thymus segments and FL-CD34^+^ and FL-CD34^−^ cells, vector transduction of FL-CD34^+^ cells, and preparation of hu-BLT mice were prepared as previously described [36] with modifications. NOD.Cg-*Prkdc^scid^ Il2rg^tm1Wjl^*/SzJ (NSG) mice were purchased from the UCLA Defined Flora Mice Facility and maintained at UCLA animal facilities. Six- to nine- week-old female NSG mice were administered Busulfan (35 mg/kg) intraperitoneally. 24 hrs later, the mice were implanted with a portion of human fetal thymus combined with FL-CD34^−^ cells and a 50∶50 mixture of control vector- and therapeutic vector- transduced FL-CD34^+^ cells solidified in Matrigel (BD Biosciences) under the kidney capsule and also transplanted with the 50∶50 vector-transduced autologous FL-CD34^+^ cell mixture via retro-orbital vein injection. To achieve high efficiencies of FL-CD34^+^ cell transduction, lentiviral vectors with minimum titers of 5×10^8^ TU/mL (in HEK-293T) were employed and desired transduction efficiencies in FL-CD34^+^ were pre-determined by transductions with titrated amounts of vector. Single-cell suspensions were prepared from peripheral blood, bone marrow, spleen, thy/liv implant, and GALT as previously described [36]. Vector-transduced hu-BLT mice were challenged with either HIV-1_NFNSX_ or HIV-1_NL4-3_, (200 ng/p24) via retro-orbital vein injection.

### Flow Cytometry

Cell staining with monoclonal antibodies and flow cytometry analysis were performed as previously described [36] with modifications. Human-specific monoclonal antibodies used were anti-CCR5 conjugated with PE-Cy7 (2D7; BD Pharmingen, San Jose, CA) or APC (2D7/CCR5; BD Pharmingen), anti-CD45 conjugated with eFluor450 (HI30; eBioscience, San Diego, CA), anti-CD3 conjugated with APC-H7 (SK7; BD Pharmingen), anti-CD19 conjugated with V500 (HIB19; BD Biosciences, San Jose, CA), CD4 conjugated with APC (OKT4; eBioscience), CD8 conjugated with PerCP-Cy5.5 (SK1; Biolegend, San Diego, CA). Murine HSA was tagged with anti-CD24 conjugated with PE-Cy7 (M1/69; BD Pharmingen). EGFP, mCherry, and antibody-tagged receptor expression were assessed using LSRFortessa^TM^ Cell Analyzer (BD Biosciences) and Cytomic FC500 (Beckman Coulter, Brea, CA).

### Cytotoxicity, Interferon Response, and Luciferase Luminescence Assays

Cytotox-Glo Cytotoxicity Assays (Promega, Madison, WI) were performed according to manufacturer's instructions. To assess induction of interferon response, total RNA from vector-transduced PBMCs was isolated using the mirVana miRNA Isolation Kit (Life Technologies). Ten ng of total RNA were subjected to OAS1 mRNA detection using the Interferon Response Detection Kit (System Biosciences, Mountain View, CA), the iScript One-Step RT-PCR Kit with SYBR Green (Bio-Rad Laboratories, Hercules, CA), and the CFX96 Real-Time PCR Detection System (Bio-Rad Laboratories) per manufacturers' instructions. OAS1 mRNA levels relative to β-globin were calculated by the ΔΔCt method. Cytotoxicity and interferon response assays were performed in triplicate. Luciferase luminescence assays were performed using the Luciferase Assay System (Promega) and the FLUOstar Optima microplate reader (BMG Labtech, Ortenbert, Germany) according to manufacturers' instructions.

### siRNA Quantitation

Total RNA from PBMCs harvested four days post-transduction or from mouse tissue-derived lymphocytes isolated eleven weeks post transplantation were isolated using the mirVana miRNA Isolation Kit (Life Technologies). cDNA synthesis was performed using 20 ng (PBMC-derived) or 10 ng (tissue-derived) of total RNA, target-specific reverse transcription primers, and the Taqman® MicroRNA Reverse Transcription Kit (Life Technologies) per manufacturer's instructions. qRT-PCR was then performed using ∼1.33 ng (PBMC-derived) or ∼0.67 ng (tissue-derived) of cDNA, target-specific FAM-based Taqman® probes, iQ^TM^ Supermix (Bio-Rad Laboratories), the CFX96 Real-Time PCR Detection System (Bio-Rad Laboratories), and the following PCR parameters: 95°C, 10 min, 1 cycle; 95°C, 15 sec and 60°C 1 min, 40 cycles. siRNA-specific RT primers and Taqman® probes were provided by Custom Taqman® Small RNA Assays (Life Technologies) generated with the following submitted target sequences:

5′-GGUGUAAACUGAGCUUGCUCUU-3′ for si1005 and 5′-GCUUUAUUGAGGCUUAAGCUU-3′ for si516. RNU38B levels were assessed by RNU38B-specific TaqMan® MicroRNA Assay (Life Technologies) for normalization of siRNA copies. Complementary pairs of synthetic RNAs (GR13-GR18, sequences found in Table S3) were annealed and serially diluted to generate quantitation standards.

### Integrated Vector Copy Quantitation

Genomic DNA from PBMCs harvested seven days post-transduction or from mouse tissue-derived lymphocytes isolated eleven weeks post-transplantation were isolated using the PureLink Genomic DNA Mini Kit (Life Technologies). Quantitative real-time DNA PCR detecting WPRE (for PBMCs), cPPT-H1 (for tissue-derived lymphocytes), and β-globin was performed using GR19-GR24 forward/reverse primers, target-specific PrimeTime^TM^ probes (Integrated DNA Technologies, Coralville, IA) (GR25-GR27), Brilliant® II QPRC Master Mix (Agilent Technologies), the Stratagene Mx300P/Mx3005P QPCR System Instrument (Agilent Technologies), and the following PCR parameters: 50°C, 2 min, 1 cycle; 95°C, 10 min, 1 cycle; 95°C, 15 sec then 60°C, 1 min, 40 cycles. Serial dilutions of FG12 plasmid, FG12 Mono sh1005 plasmid, and PBMC-derived genomic DNA were used to generate quantitation standards for WPRE, cPPT-H1, and β-globin copies, respectively. Sequences of primers and probes used for qPCR analysis can be found in Table S3.

### Colony Formation Assay

Vector-transduced mPB-CD34^+^ cells were resuspended in MethoCult H4034 (Stem Cell Technologies, Vancouver, Canada) and 500 cells were plated per replicate. Twelve to fourteen days later, CFUs were counted and scored using a Nikon Eclipse TS100 (Nikon, Melville, NY) microscope.

## Supporting Information

Figure S1 Optimization of sh516-expressing vectors via mutagenesis of vector LTRs. **A.** Schematic of potential RNAi-mediated attenuation of sh1005-sh516 vector anti-viral activity. The sh516 target sequence (black box) resides within the LTR R region as well as the sh516 expression cassette. The sh1005 expression cassette possesses an sh1005 target sequence (striped box). sh516 may target vector LTRs in packaging cell as well as vector–derived mRNA, reducing vector titer and protein expression. **B.** Schematic of the sh516 target sequence and vector LTR mutation. **C.** Vector titers of unconcentrated vector stocks were calculated by transduction of HEK-293T cells. Titers were normalized by p24 concentration of vector preparation. **D.** Marker gene expression in HEK-293T cells transduced with lentiviral vectors. mCherry MFI in mCherry^+^ cells was assessed by flow cytometry analysis. Parental –sh1005/sh516 co-expressing vector. LTR modified – sh1005/sh516 co-expressing vector with modified vector LTRs. (**C–D**) Error bars – mean + SD. Statistical significance calculated by Student *t* test. ns  =  not statistically significant. ***  =  p value ≤0.0002.
(TIF)

Table S1 siRNA expression in vector-transduced hematopoietic cells.
(DOCX)

Table S2 Gene-marked CD4^+^ T-cell levels in peripheral blood of HIV-1-infected Dual sh1005/sh516-transduced hu-BLT mice.
(DOCX)

Table S3 Sequences of DNA and RNA oligos used in this study.
(DOCX)
