# Supplementary figures and images for: Engineering HIV-1-Resistant T-Cells from Short-Hairpin RNA-Expressing Hematopoietic Stem/Progenitor Cells in Humanized BLT Mice
Source: PLoS One. 2012 Dec 31;7(12):e53492. doi: 10.1371/journal.pone.0053492 (PMC3534037; doi:10.1371/journal.pone.0053492)

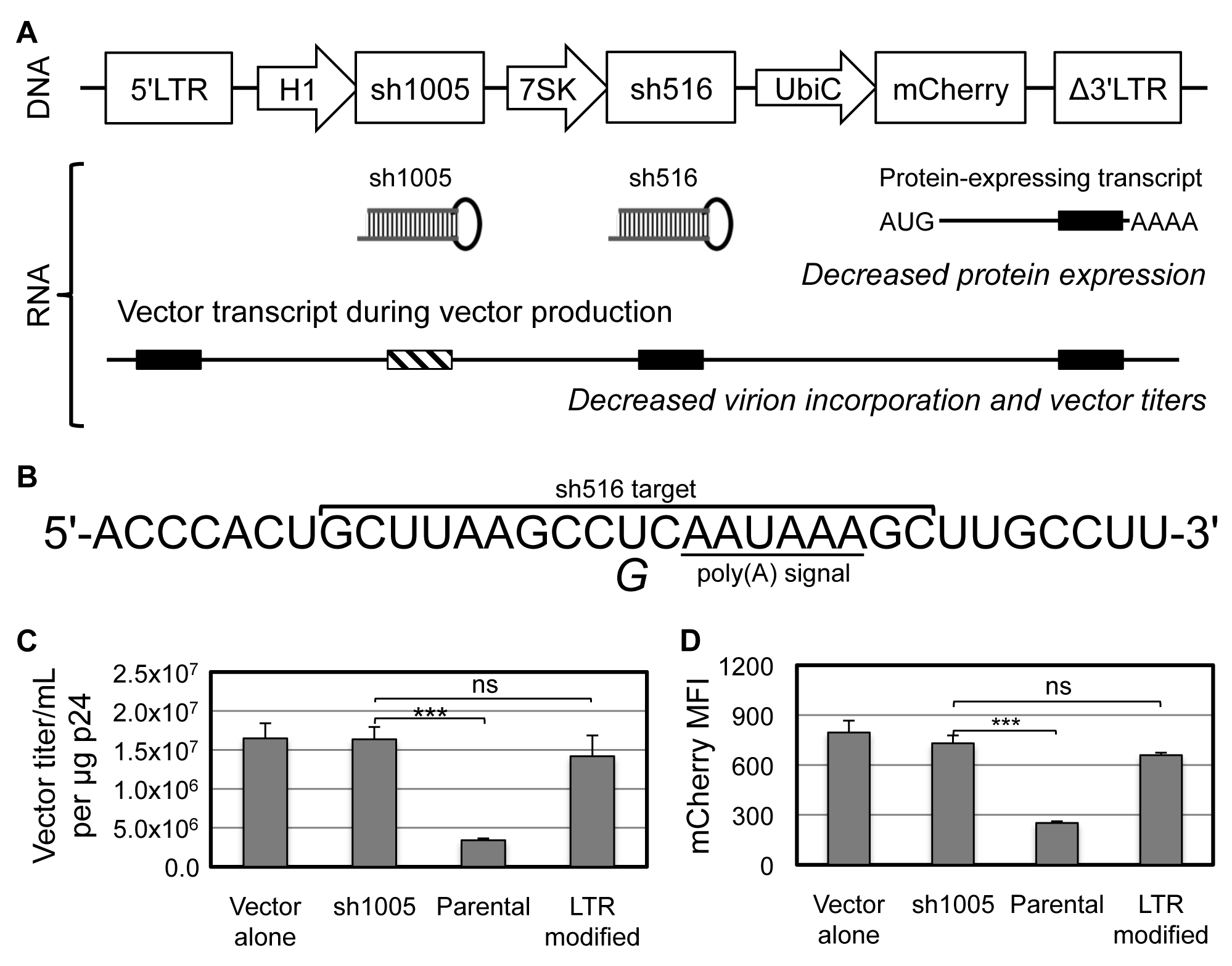

Supplement: Figure S1 — Optimization of sh516-expressing vectors via mutagenesis of vector LTRs. A. Schematic of potential RNAi-mediated attenuation of sh1005-sh516 vector anti-viral activity. The sh516 target sequence (black box) resides within the LTR R region as well as the sh516 expression cassette. The sh1005 expression cassette possesses an sh1005 target sequence (striped box). sh516 may target vector LTRs in packaging cell as well as vector–derived mRNA, reducing vector titer and protein expression. B. Schematic of the sh516 target sequence and vector LTR mutation. C. Vector titers of unconcentrated vector stocks were calculated by transduction of HEK-293T cells. Titers were normalized by p24 concentration of vector preparation. D. Marker gene expression in HEK-293T cells transduced with lentiviral vectors. mCherry MFI in mCherry+ cells was assessed by flow cytometry analysis. Parental –sh1005/sh516 co-expressing vector. LTR modified – sh1005/sh516 co-expressing vector with modified vector LTRs. (C–D) Error bars – mean + SD. Statistical significance calculated by Student t test. ns = not statistically significant. *** = p value ≤0.0002. (TIF) [file pone.0053492.s001.tif]
