# Supplementary material for: Engineering HIV-1-Resistant T-Cells from Short-Hairpin RNA-Expressing Hematopoietic Stem/Progenitor Cells in Humanized BLT Mice
Source: PLoS One. 2012 Dec 31;7(12):e53492. doi: 10.1371/journal.pone.0053492 (PMC3534037; doi:10.1371/journal.pone.0053492)
Supplement: Table S1 — siRNA expression in vector-transduced hematopoietic cells. (DOCX) [file pone.0053492.s002.docx]

| Sample | %EGFP+ | Integrated vector copies^a^ | | RNA copies^c^ | | | | Normalized siRNA expression per transduced cell^d^ | |
| --- | --- | --- | --- | --- | --- | --- | --- | --- | --- |
|  |  | TOTAL^a^ | Transduced^b^ | si516 | RNU38B (si516 control) | si1005 | RNU38B (si1005 control) | si516 | si1005 |
| In vitro-stimulated vector-transduced peripheral blood mononuclear cells^e^ | | | | | | | | | |
| Mock | 1.2 | 0.0 ± 0.0 | 0.00 | ND | 9.8x10^3^ ± 3.1x10^2^ | ND | 9.7x10^3^ ± 3.1x10^2^ | ND | ND |
| Vector alone | 26.7 | 0.28 ± 0.03 | 1.06 | ND | 1.1x10^4^ ± 2.2x10^2^ | ND | 1.1x10^4^ ± 2.2x10^2^ | ND | ND |
| Mono sh1005 | 21.5 | 0.24 ± 0.02 | 1.13 | ND | 1.3x10^4^ ± 3.5x10^2^ | 1.5x10^5^ ± 1.2x10^4^ | 1.3x10^4^ ± 3.5x10^2^ | ND | 10.4 |
| Mono sh516 | 19.5 | 0.20 ± 0.01 | 1.01 | 2.4x10^4^ ± 9.7x10^2^ | 1.5x10^4^ ± 1.4x10^3^ | ND | 1.5x10^4^ ± 1.4x10^3^ | 1.6 | 0.0 |
| Dual sh1005/sh516 | 19.1 | 0.14 ± 0.01 | 0.74 | 2.4x10^4^ ± 5.7x10^2^ | 1.0x10^4^ ± 1.8x10^2^ | 5.9x10^4^ ± 5.1x10^3^ | 1.0x10^4^ ± 1.8x10^2^ | 3.1 | 7.7 |
| Dual sh516-transduced BLT mouse tissue-derived lymphocytes^f^ | | | | | | | | | |
| Bone marrow | 14.3 | 0.62 ± 0.03 | 4.34 | 5.2x10^3^ ± 9.7x10^2^ | 5.3x10^4^ ± 4.0x10^3^ | 2.8x10^4^ ± 2.6x10^3^ | 5.9x10^4^ ± 1.9x10^3^ | 0.02 | 0.11 |
| Spleen | 16.1 | 0.59 ± 0.01 | 3.66 | 6.2x10^3^ ± 4.3x10^2^ | 3.9x10^4^ ± 4.9x10^3^ | 2.4x10^4^ ± 1.2x10^3^ | 4.8x10^4^ ± 1.1x10^3^ | 0.04 | 0.14 |

**Table S1.** siRNA expression in vector-transduced hematopoietic cells.

^a^Average and standard deviation of integrated vector copies were calculated from duplicate samples.

^b^Integrated vector copies per transduced cell calculated by [Integrated vector copies: TOTAL] × 100 ÷ [%EGFP+].

^c^Average and standard deviation of si516, si1005, and RNU38B copies were calculated from triplicate samples. ND = not detected.

^d^Normalized siRNA expression per transduced cell calculated by [RNA copies: siRNA] ÷ [RNA copies: RNU38B] ÷ [Integrated vector copies: Transduced].

^e^IL-2/PHA-stimulated PBMCs were transduced with lentiviral vectors at MOI 0.6-1.0.

^f^Lymphocytes were isolated from tissues derived from a BLT mouse transplanted with FL-CD34+ cells vector-transduced with 69.4% efficiency.
