# Supplementary material for: Engineering HIV-1-Resistant T-Cells from Short-Hairpin RNA-Expressing Hematopoietic Stem/Progenitor Cells in Humanized BLT Mice
Source: PLoS One. 2012 Dec 31;7(12):e53492. doi: 10.1371/journal.pone.0053492 (PMC3534037; doi:10.1371/journal.pone.0053492)
Supplement: Table S2 — Gene-marked CD4+ T-cell levels in peripheral blood of HIV-1-infected Dual sh1005/sh516-transduced hu-BLT mice. (DOCX) [file pone.0053492.s003.docx]

**Table S2.** Gene-marked CD4^+^ T-cell levels in peripheral blood of HIV-1-infected Dual sh1005/sh516-transduced hu-BLT mice.

| HIV-1_NFNSX_-infected | | | | | | | | | | | | | | |
| --- | --- | --- | --- | --- | --- | --- | --- | --- | --- | --- | --- | --- | --- | --- |
|  | % marked CD4^+^ in total CD3^+^ | | | | | | | | | | | |  | |
|  | Pre-infection | | Week 4 (Early) | | Week 6 | | Week 8 | | Week 10 | | Week 12 (Late) | | Percent change in CD4^+^ in CD3^+a^ | |
| Mouse | mCh | EGFP | mCh | EGFP | mCh | EGFP | mCh | EGFP | mCh | EGFP | mCh | EGFP | mCh | EGFP |
| 844 | 17.2 | 7.41 | 24.0 | 46.5 | 10.1 | 45.4 | 7.1 | 61.9 | 3.7 | 69.1 | 1.8 | 69.7 | -92 | +50 |
| 846 | 23.9 | 15.4 | 52.0 | 6.4 | 41.3 | 10.5 | 48.5 | 12.4 | 44.3 | 16.1 | 42.1 | 16.2 | -19 | +152 |
| 848 | 29.9 | 11.8 | 36.5 | 8.1 | 29.4 | 11.2 | 32.5 | 11.8 | 29.4 | 12.6 | 13.3 | 9.4 | -64 | +16 |
| 856 | 28.2 | 12 | 34.0 | 8.0 | 26.7 | 8.9 | 29.1 | 9.9 | 23.5 | 14.2 | 12.7 | 10.6 | -63 | +33 |
| 861 | 34.3 | 10.9 | 41.3 | 11.3 | 24.4 | 19.8 | 23.4 | 29.0 | 16.4 | 36.5 | 12.6 | 35.8 | -69 | +217 |
| HIV-1_NL4-3_-infected | | | | | | | | | | | | | | |
|  | % marked CD4^+^ in total CD3^+^ | | | | | | | | | | | |  | |
|  | Pre-infection | | Week 2 (Early) | | Week 4 | | Week 6 | | Week 8 | | Week 10 (Late) | | Percent change in CD4^+^ in CD3^+^ | |
| Mouse | mCh | EGFP | mCh | EGFP | mCh | EGFP | mCh | EGFP | mCh | EGFP | mCh | EGFP | mCh | EGFP |
| 843 | 27.4 | 14.2 | 42.9 | 5.7 | 34.6 | 10.8 | 30.1 | 11.9 | 28.7 | 11.3 | 22.8 | 11.0 | -47 | +92 |
| 847 | 38.7 | 12.2 | 51.9 | 3.6 | 37.3 | 4.4 | 43.9 | 3.9 | 41.9 | 3.0 | 30.6 | 3.5 | -41 | -1 |
| 849 | 31.0 | 7.83 | 25.6 | 14.9 | 16.8 | 16.8 | 19.4 | 17.0 | 18.5 | 18.1 | 16.2 | 17.2 | -37 | +16 |
| 857 | 34.3 | 8.87 | 39.4 | 3.4 | 29.5 | 5.3 | 31.5 | 5.7 | 23.9 | 4.4 | 18.6 | 4.3 | -53 | +27 |

^a^Percent change in marked CD4^+^ cells in total CD3^+^ cells was calculated as ([Late] – [Early]) ÷ [Early] × 100. Positive and negative values show increase and decrease, respectively, in percent change.
