# Supplementary material for: Engineering HIV-1-Resistant T-Cells from Short-Hairpin RNA-Expressing Hematopoietic Stem/Progenitor Cells in Humanized BLT Mice
Source: PLoS One. 2012 Dec 31;7(12):e53492. doi: 10.1371/journal.pone.0053492 (PMC3534037; doi:10.1371/journal.pone.0053492)
Supplement: Table S3 — Sequences of DNA and RNA oligos used in this study. (DOCX) [file pone.0053492.s004.docx]

**Table S3.** Sequences of DNA and RNA oligos used in this study.

| **Name** | **Sequence (5'-3')** | **Function** |
| --- | --- | --- |
| **shRNA expression** | | |
| GR01 | GCTGCTCGAGAAAAAGCATCTCCTATGGCAGGAAGTCGGACAATTCCTGCCATAGGAGATGCGGGGATCCGTGGTCTCATAC | H1-sh131 (A) |
| GR02 | GCTGGTCGACAAAAAGAGCAGATGATACAGTATTtctcgagtAATACTGTATCATCTGCTCGGGGATCCGTGGTCTCATAC | H1-sh248 (A) |
| GR03 | GCTGCTCGAGAAAAACCCACTGCTTAAGCCTCAGTCGGACAATGAGGCTTAAGCAGTGGGTGGGGATCCGTGGTCTCATAC | H1-sh509 (A) |
| GR04 | GCTGCTCGAGAAAAAGCTTAAGCCTCAATAAAGCGTCGGACAAGCTTTATTGAGGCTTAAGCGGGGATCCGTGGTCTCATAC | H1-sh516 (A) |
| GR05 | GCTGCTCGAGAAAAAGCCTCAATAAAGCTTGCCTGTCGGACAAAGGCAAGCTTTATTGAGGCGGGGATCCGTGGTCTCATAC | H1-sh522 (A) |
| GR06 | GCTGCTCGAGAAAAATAAAGCTTGCCTTGAGTGTCGGACAAACTCAAGGCAAGCTTTATTGGGGATCCGTGGTCTCATAC | H1-sh527 (A) |
| GR07 | GCTGGTCGACAAAAAGGAGCCACCCCACAAGATTtctcgagtAATCTTGTGGGGTGGCTCCGGGGATCCGTGGTCTCATAC | H1-sh532 (A) |
| GR08 | GCTGGTCGACAAAAACAATTGGAGAAGTGAATTtctcgagtAATTCACTTCTCCAATTGTGGGGATCCGTGGTCTCATAC | H1-sh1424 (A) |
| GR09 | GCTGCTCGAGAAAAAGGCTCCTCAGAAACAGCTCGTCGGACAAGAGCTGTTTCTGAGGAGCCGGGGATCCGTGGTCTCATAC | H1-shLuc (A) |
| GR10 | ACCTCGCTTAAGCCTCAATAAAGCTTGTCCGACGCTTTATTGAGGCTTAAGCTT | 7SK-sh516 (S) |
| GR11 | GAAAAAGCTTAAGCCTCAATAAAGCGTCGGACAAGCTTTATTGAGGCTTAAGCG | 7SK-sh516 (A) |
| **Mutagenesis** | | |
| GR12 | gaacccactgcttaagccgcaataaagcttgccttg | Vector LTR |
| **Synthetic RNA for qRT-PCR standards** | | |
| GR13 | GCUUUAUUGAGGCUUAAGC(dT)(dT) | si516 (S) |
| GR14 | GCUUAAGCCUCAAUAAAGC(dT)(dT) | si516 (A) |
| GR15 | GGUGUAAACUGAGCUUGCUC(dT)(dT) | si1005 (S) |
| GR16 | GUGCUUGCTCUGTTTUCUCC(dT)(dT) | si1005 (A) |
| GR17 | CCAGUUCUGCUACUGACAGUAAGUGAAGAUAAAGUGUGUCUGAGGAGA | RNU38B (S) |
| GR18 | UCUCCUCAGACACACUUUAUCUUCACUUACUGUCAGUAGCAGAACUGG | RNU38B (A) |
| **DNA qPCR** | | |
| GR19 | CCGTTGTCAGGCAACGTG | WPRE amp (S) |
| GR20 | AGCTGACAGGTGGTGGCAAT | WPRE amp (A) |
| GR21 | GGGTTTATTACAGGGACAGCAG | cPPT-H1 (S) |
| GR22 | ATTCCTTGGAGCGGGTTG | cPPT-H1 (A) |
| GR23 | CAACCTCAAACAGACACCATGG | Beta globin amp (S) |
| GR24 | TCCACGTTCACCTTGCCC | Beta globin amp (A) |
| GR25 | 6-FAM/TGCTGACGCAACCCCCACTGGT/3BHQ_1 | WPRE Taqman |
| GR26 | 6-FAM/CGTCAGCGT/ZEN/TCGAATTCCATGGTCT/3IABkFQ | cPPT-H1 Taqman |
| GR27 | 5TexRd-XN/CTCCTGAGGAGAAGTCTGCCGTTACTG CC/3BHQ_2 | Beta globin Taqman |

(S): sense amp: PCR amplification 6-FAM: 5' 6-FAM^TM^ 3BHQ_1: 3' Black Hole Quencher® 1

(A): antisense (dT): deoxythymydine 5TexRd-XN: 5' Texas Red®-X (NHS Ester) 3BHQ_2: 3' Black Hole Quencher® 2 ZEN…3IABkFQ: ZEN/Iowa Black FQ
